# Bracing for juvenile idiopathic scoliosis: retrospective review from bracing to skeletal maturity

**DOI:** 10.1007/s43390-022-00544-2

**Published:** 2022-07-19

**Authors:** Amanda T. Whitaker, Michael Timothy Hresko, Patricia E. Miller, Bram P. Verhofste, Alexandra Beling, John B. Emans, Lawrence I. Karlin, Daniel J. Hedequist, Michael P. Glotzbecker

**Affiliations:** 1 Department of Orthopaedic Surgery, Shriners Hospital for Children Northern California, 2425 Stockton Blvd, Sacramento, CA 95817 USA; 2 Department of Orthopaedic Surgery, University of California Davis, 4680 Y St, Sacramento, CA 95817 USA; 3 Department of Orthopedic Surgery, Boston Children’s Hospital, 300 Longwood Ave HU 221, Boston, MA 02115 USA; 4 Department of Orthopedic Surgery, Rainbow Babies and Children’s Hospital, 11100 Euclid Ave, Cleveland, OH 44106 USA

**Keywords:** Juvenile idiopathic scoliosis, Scoliosis, Bracing, Non-operative treatment scoliosis, Spinal fusion

## Abstract

**Background:**

Juvenile idiopathic scoliosis (JIS) outcomes with brace treatment are limited with poorly described bracing protocols. Between 49 and 100% of children with JIS will progress to surgery, however, young age, long follow-up, and varying treatment methods make studying this population difficult. The purpose of this study is to report the outcomes of bracing in JIS treated with a Boston brace™ and identify risk factors for progression and surgical intervention.

**Methods:**

This is a single-center retrospective review of 175 patients with JIS who initiated brace treatment between the age of 4 and 9 years. A cohort of 140 children reached skeletal maturity; 91 children had surgery or at least 2 year follow-up after brace completion. Standard in-brace protocol for scoliosis ^3^20° was a Boston brace for 18–20 h/day after MRI (*n* = 82). Family history, MRI abnormalities, comorbidities, curve type, curve magnitude, bracing duration, number of braces, compliance by report, and surgical interventions were recorded.

**Results:**

Children were average 7.9 years old (range 4.1–9.8) at the initiation of bracing. The Boston brace™ was prescribed in 82 patients and nine used night bending brace. Mid-thoracic curves (53%) was the most frequent deformity. Maximum curve at presentation was on average 30 ± 9 degrees, in-brace curve angle was 16 ± 8 degrees, and in-brace correction was 58 ± 24 percent. Patients were braced an average of 4.6 ± 1.9 years. 61/91 (67%) went on to posterior spinal fusion at 13.3 ± 2.1 (range 9.3–20.9) years and curve magnitude of 61 ± 12 degrees. Of those that underwent surgery, 49/55 (86%) progressed > 10°, 6/55 (11%) stabilized within 10°, and 0/55 (0%) improved > 10° with brace wear. No children underwent growth-friendly posterior instrumentation. Of the 28 who did not have surgical correction, 3 (11%) progressed > 10°, 13/28 (46%) stabilized within 10°, and 12/28 (43%) improved > 10° with brace wear.

**Conclusions:**

This large series of JIS patients with bracing followed to skeletal maturity with long-term follow-up. Surgery was avoided in 33% of children with minimal to no progression, and no child underwent posterior growth-friendly constructs. Risk factors of needing surgery were noncompliance and larger curves at presentation.

## Introduction

Idiopathic scoliosis (IS) in children is defined as a spinal curvature ^3^10° without an identifiable etiology such a congenital malformation or neuromuscular disorder [1, 2]. Juvenile idiopathic scoliosis (JIS) includes patients with onset specifically between 4 and 10 years old and is a subset of early-onset scoliosis (EOS), which describes any scoliosis < 10 years of age [3–7]. JIS differs from adolescent idiopathic scoliosis (AIS) as it is less common, the rate of curve progression is greater, and the incidence of surgical intervention is higher (41–100%) [3–6, 8–14]. Associated intraspinal pathologies are often found in children with EOS, with incidences ranging between 11 and 26%, and increased mortality is reported [12, 15–18].

Previous studies have attempted to address the effectiveness of bracing in JIS [4, 8, 10, 11, 19]. These studies are often limited due to mixed methodologies resulting from variability in presenting curve magnitudes, age at bracing, brace type, brace-wear duration, definition of successful outcomes, surgical thresholds, MRI data, follow-up length, and study periods that span several decades with different treatment protocols. Young age, long follow-up, and varying treatment methods make studying this population difficult.

The purpose of this study was to present the experience of a large tertiary center treating JIS with a standard bracing protocol until skeletal maturity with long-term follow-up and outcomes in accordance with the Scoliosis Research Society (SRS) recommendations [9].

## Materials and methods

**Fig. 1:**
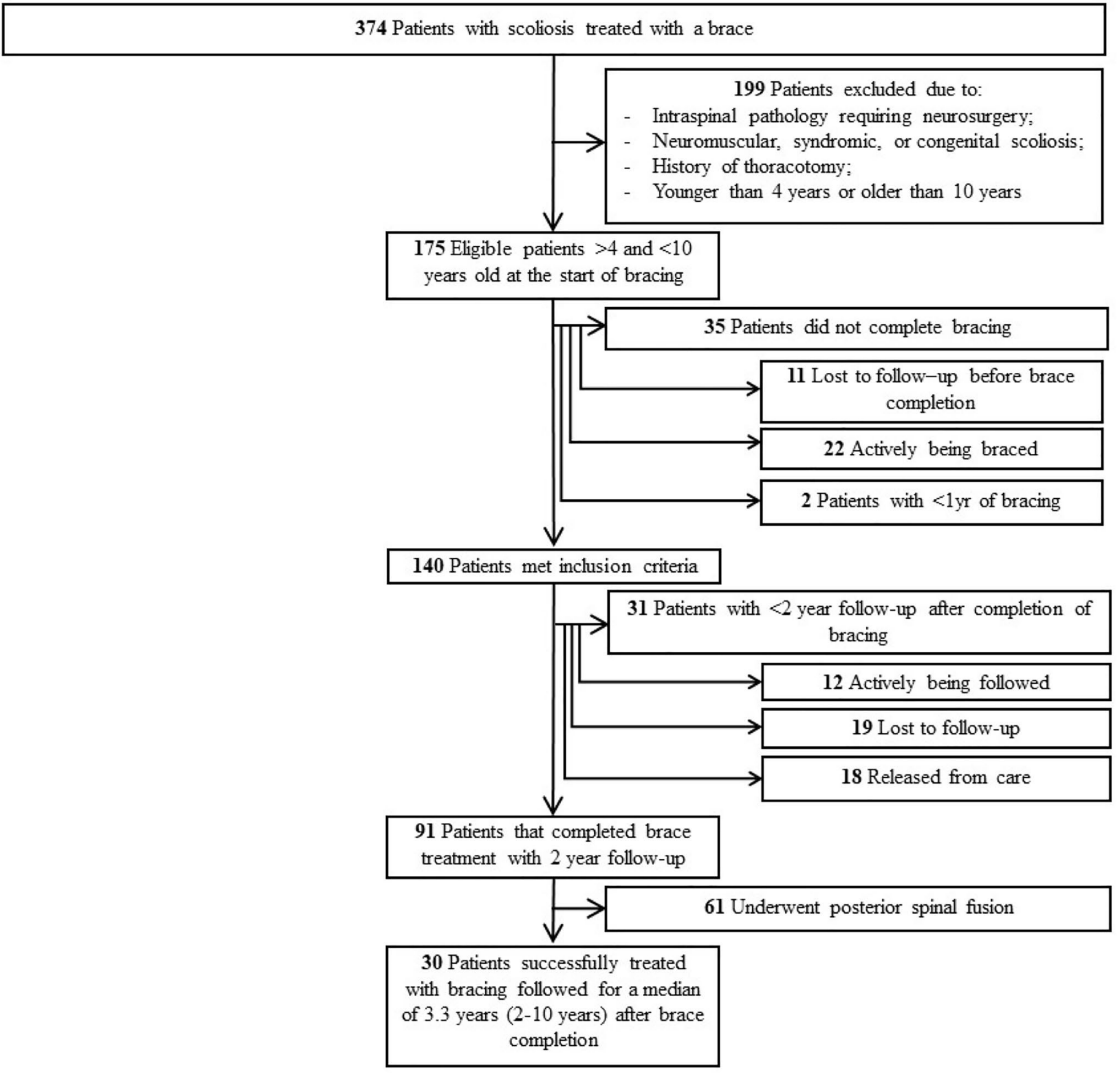
Flowchart of study inclusion

A retrospective review of 374 consecutive children between the ages of 4 and 10 years treated with a brace for scoliosis during 2002–2012 was performed. Twenty-three cases were excluded for intraspinal pathology (ISP) that required neurosurgical intervention. Other exclusion criteria included: non-idiopathic etiologies of scoliosis (neuromuscular, syndromic, or congenital), previous thoracotomy, advanced bone age at brace initiation, children braced $\ge$ 10 or $<$ 4 years old, or children in a brace < 1 year prior to surgery. One hundred and seventy-five JIS patients treated with a thoracolumbar sacral orthosis were identified (Fig. 1). At the completion of bracing, 140 children (80%) had reached skeletal maturity or underwent spinal fusion after wearing a brace for at least 1 year. Of those who had not undergone surgical intervention, 12 (9%) were within 2 years after brace completion and 19 (14%) were lost to follow-up. Of the 109 remaining patients, 61 underwent surgical intervention, 18 were released from care at the completion of bracing, and 30 had greater than 2 year follow-up median of 3.3 years (range 2–10). Thus, 91 subjects were analyzed for outcomes following bracing protocol. All components of this study were approved by the Institutional Review Board.

Sex, BMI, age, family history, MRI results, comorbidities, curve type, Cobb angle, rib-vertebral angle difference (RVAD), brace type, in-brace time prescribed, bracing duration, and number of brace-type were recorded.

**Fig. 2:**
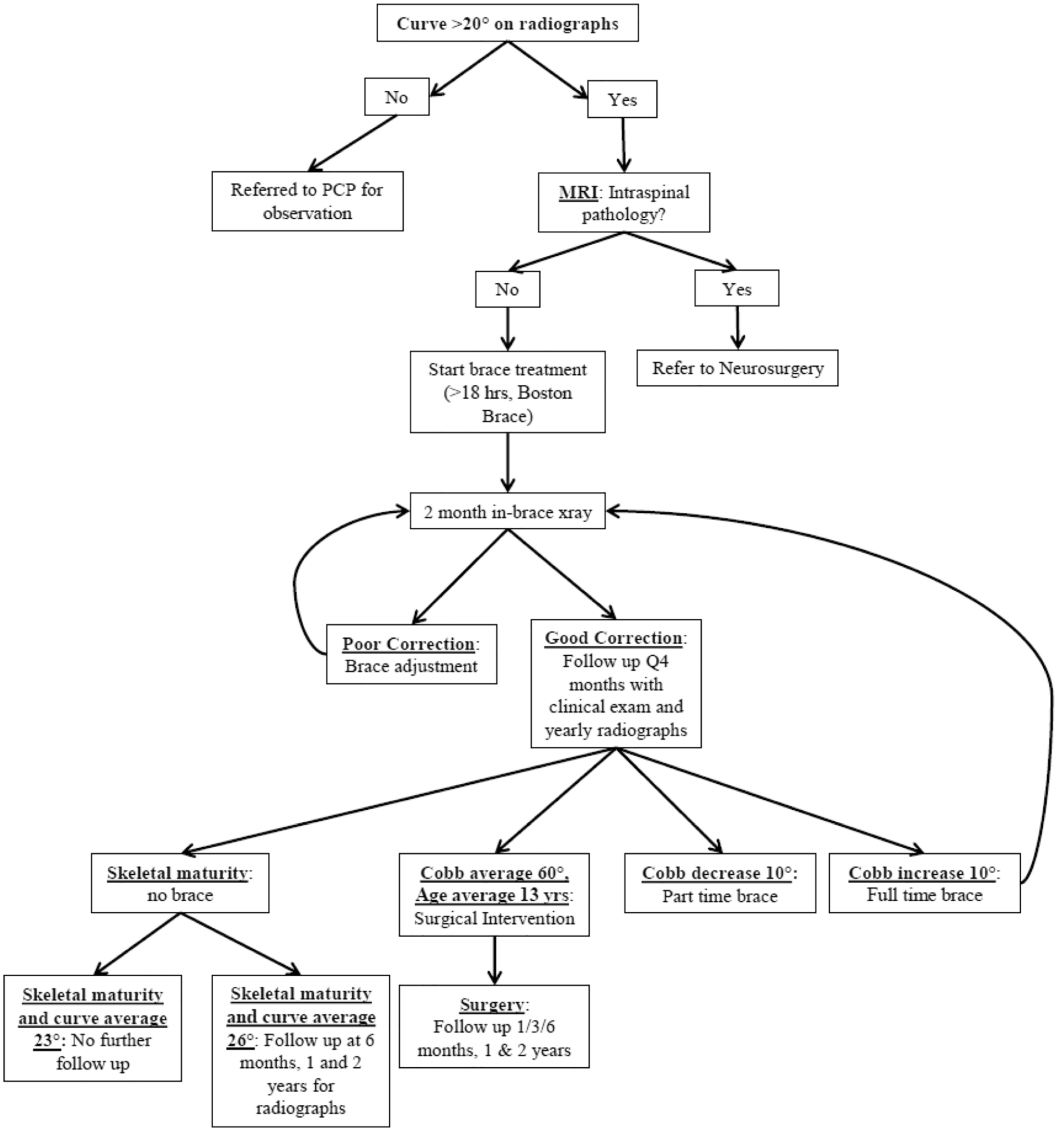
Diagram of standard treatment of JIS scoliosis at our institution

The standard evaluation of a child between 4 and 10 years old presenting with scoliosis $\ge$ 20° at our institution included an MRI to evaluate for intraspinal pathology (Fig. 2). The MRI was abnormal but did not require intervention in 19/83 patients (23%). These abnormalities included syrinx not associated with Chiari malformation, fatty filum, prominent central canal, low lying conus, cisterna cyst, spondylolisthesis, renal anomaly, thyroid mass, and thoracic compression fracture (Table 1). The 14 children (15%) with intraspinal pathology (ISP) not requiring intervention were braced.

**Table 1:** Patient and MRI characteristics (*n* = 91)

Characteristic	Freq.	(%)	(Range)
Age at presentation (years; mean ± SD)	7.6	± 1.5	(4.0–9.8)
Sex (% female)	8	(9%)	
Height (cm; mean ± SD; *n* = 90)*	130	± 10.8	(100.5–169.0)
Weight (kg; mean ± SD; *n* = 89)*	29.3	± 10.2	(13.0–81.5)
BMI percentile (mean ± SD; *n* = 89)*	57.3	± 29.4	(2.4–99.8)
Obese	12	(14%)	
Overweight	8	(9%)	
Normal weight	67	(75%)	
Underweight	2	(2%)	
Comorbidity (patients with ≥ 1 comorbidity)	35	(39%)	
Leg limb discrepancy	10	(11%)	
Neurologic	6	(7%)	
Spondylolisthesis	1	(1%)	
Cardiac	3	(3%)	
Developmental dysplasia of hip	10	(11%)	
Other orthopaedic^a^	5	(6%)	
Respiratory	2	(2%)	
Gastrointestinal	2	(2%)	
Renal	1	(1%)	

MRI Characteristics	Freq.	(%)	
Abnormal MRI (*n* = 83)*	19	(23%)	
Intraspinal pathology	14	(15%)	
Syrinx	5	(5%)	
Fatty filum	3	(3%)	
Prominent central canal	2	(2%)	
Low conus	2	(2%)	
Hydrosyringomyelia	1	(1%)	
Vascular anomaly	1	(1%)	
Other	5	(5%)	
Spondylolisthesis	2	(2%)	
Renal anomaly	1	(1%)	
Thyroid mass	1	(1%)	
Lumbar facet arthropathy	1	(1%)	

*The number in parentheses (*n*) indicates the number of patients with available data for the given characteristic

*SD* standard deviation, *BMI* body mass index

^a^1 case of Osgood-Schlatters diease and 1 case of osteopenia

^b^Neurologic comorbidities included: epilepsy (*n* = 3), autism spectrum disorder (*n* = 3), conductive hearing loss (*n* = 1), depression / anxiety (*n* = 1), Duane syndrome (*n* = 1), and epidural hematoma (*n* = 1)

### Brace protocol

The Boston brace was prescribed in 82 patients (90%) and the Charleston night brace in 9 patients based on physician/patient/parent discussion (10%) (Table 2). Full time bracing (≥ 18 h/day) was prescribed for patient in the Boston Brace for the initial brace in 75 of 82 cases (82%), with night time wear for nine patients in Charleston bracing. All patients had full-time bracing during their course of treatment. Compliance was determined using physician’s progress notes based on family reported compliance.as compliance monitors were not available at that time. [4, 11, 20]

**Table 2:** Curve and bracing characteristics (*n* = 91)

Initial curve characteristics	Freq.	(%)	
Curve type			
Mid-thoracic	48	(5%)	
Double major	16	(18%)	
Thoracolumbar / lumbar	16	(18%)	
Double thoracic	11	(12%)	
Triple major	0	(0%)	

Initial curve magnitude*	Mean	± SD	(Range)
Proximal thoracic (°)	14.0	± 7.43	(0–33)
Mid-thoracic (°)	28.1	± 10.11	(1–50)
Thoracolumbar / lumbar (°)	20.4	± 8.73	(0–43)
Maximum curve magnitude (°)	29.9	± 8.62	(12–50)
Maximum curve type (freq. (%))			
Proximal thoracic	2	(2%)	
Mid-thoracic	67	(74%)	
Thoracolumbar / lumbar	14	(15%)	
Maximum curve ≥ 30° (freq. (%))	30	(36%)	
RVAD (*n* = 80; °)*	8.6	± 7.77	0–38

Bracing characteristics	Freq.	(%)	(Range)
Age at brace initiation (years; median(IQR))	7.9	± 1.41	(4.1–9.8)
Number braces (median (IQR))	3	2–3	(1–6)
Boston brace prescribed	82	(90%)	
Non-compliant	53	(58%)	
Brace-wear hours (median (IQR))	18	18–20	(8–23)
Bracing duration (years; mean ± SD)	4.6	± 1.93	(1.0–9.7)
Maximal curve magnitude in-brace (*n* = 87; °)*	15.6	± 7.66	(2–39)
Percent in-brace correction (*n* = 81; %)	58.3	± 23.97	(− 3.4–100.0)

Surgical intervention	Mean	± SD	(Range)
Progression to surgery (freq. (%))	61	(67%)	
Age at surgery (years)	13.3	± 2.15	(9.3–20.9)
Final curve magnitude (°)	60.6	± 12.10	(33–93)

^*^The number in parentheses (*n* =) indicates the number of patients with available data for the given characteristic

*SD* standard deviation, *IQR* interquartile range, *RVAD* rib-vertebral angle difference

Patients were evaluated after the brace was constructed with an in-brace radiograph. The in-brace correction goal was ≥ 50% of the major curve. If this was not achieved, the brace was adjusted to obtain maximal correction. Children were seen in clinic every four months for a clinical exam and intermittent radiographs. The recommended time in the brace was at surgeon discretion, with general principle of 18–20 h/day customized to individual patient. Skeletal maturity was defined by bone age, linear growth < 1 cm over 6 months, and Risser 4/5. Brace compliance was based on parent report and physician assessment. No data on hours of brace wear per day was obtained as brace compliance monitors were not available during the study years. The decision for brace discontinuation was made through a combination of shared decision making with the patient, parent and physician combined with skeletal maturity.

### Statistical analysis

Patient and curve characteristics were summarized for all patients in accordance with the SRS and SOSORT guidelines [9, 21]. Continuous characteristics were summarized by mean and standard deviation (SD) or median and interquartile range (IQR, 25th–75th percentile) and range; while categorical characteristics were summarized by frequency and percent. Curve magnitudes for proximal thoracic, mid-thoracic, and thoracolumbar/lumbar curves were analyzed at presentation, in-brace, completion of bracing, and preoperatively or latest follow-up when available. Maximal curves were summarized at each follow-up for the largest curve at that time.

Curve and bracing characteristics were analyzed for possible effects on surgical intervention using multivariable logistic regression. Factors included: age, comorbidities, family history, curve type, maximum curve, brace changes, percent in-brace correction, and reported compliance. Odds ratios (OR) along with 95% confidence intervals (95% CI) were estimated for significant risk factors. All tests were two-sided and type I error was set at 5%.

Patient and curve characteristics were summarized for the 18 patients who were released from care. The underlying assumption is that these patients had enough correction in their curves following treatment with bracing that further intervention was not expected to be required. To assess the influence of these subjects on study outcomes, an additional analysis was conducted that included these 18 patients in the non-surgical intervention group to assess any affect or variation in the analysis for the likelihood of surgical intervention.

## Results

### Initial evaluation

Ninety-one patients (83 females) with an average age of 7.6 ± 1.5 (range 4–9.8) years at presentation were followed for a median of 6.5 years (IQR 4.8–8.5) (Table 1). Eight girls (9%) and no boys were < 6 years old at presentation. One or more comorbidities were seen in 35 patients (39%) and included leg length discrepancy, spondylolisthesis, cardiac abnormalities, epilepsy, ADHD, and gastrointestinal disorders (Table 1).

### Bracing

At presentation, mean maximal curves were 30 ± 9 (range 12–50) degrees (Table 2). Mid-thoracic curves were the most frequent curve type at presentation with 48 cases (53%). Patients began bracing at a median age of 7.9 (4.1–9.8) years. Mean in-brace curves were 16 ± 8 (range 2–39) degrees with an average in-brace correction of 58 ± 24 (range − 3–100) percent (Table 2). Average bracing duration was 4.6 ± 1.9 (range 1.0–9.7) years with a median of three brace changes (IQR 2–3). Fifty-three patients (58%) were non-compliant with the recommended brace wear based on self-report. Mean maximum curves were 43 ± 19 (range 5–93) degrees at brace completion on standing out of brace radiograph (Table 4). Median follow-up after brace completion was 3.3 (range 2–9.4) years.

### Surgical correction

Sixty-one patients (67%) underwent surgical intervention at a mean age of 13.3 ± 2.15 years (9.3–20.9yrs) and maximum curve magnitude of 60° ± 12.10° (range 33°–93°) (Table 2). Median time from end of brace to surgery was 0.3 years (range 0–10.5 years). Posterior spinal fusion (PSF) was performed in all patients. No patients underwent growth-friendly posterior instrumentation.

Children that underwent PSF had greater curves at presentation (32° vs. 26°; *p* = 0.008), larger in-brace curves (17° vs. 12°; *p* = 0.009), and larger curves at the end of bracing (52° vs. 22°; *p* < 0.001) (Table 3). In addition, the percentage of self-reported non-compliant subjects was nearly three times higher in the PSF group (74 vs. 27%; *p* < 0.001). No difference was detected in the in-brace correction (55 vs. 64%; *p* = 0.11). Multivariable analysis determined that for each additional 1° increase in presentation curve magnitude, the odds of surgery increased by 11% (OR 1.11, 95% CI 1.04–1.19, *p* = 0.004). Patients who were non-compliant with bracing had 6.3 times the odds of PSF (OR 6.32, 95% CI 6.12–18.86, *p* < 0.001) compared to compliant patients. Those with at least one comorbid factor had 2.5 times the odds of PSF (OR 3.44, 95% CI 1.04–11.35, *p* = 0.04) compared to patients with no comorbidities. Non-operative intraspinal pathology (ISP) did not affect the surgical rate; 10/63 surgical patients had ISP (16%) and 4/30 non-op (13%) had ISP (*p* = 0.99).

**Table 3:** Patient, curve, and bracing characteristics by surgical intervention group

	Surgical intervention (*n* = 61)		Conservative management (*n* = 30)		
Presentation characteristics	Freq.	(%)	Freq.	(%)	*p*
Age at presentation (years; mean ± SD)	7.5	± 1.3	7.8	± 1.7	0.49
Sex (% female)	4	(7%)	4	(13%)	0.29
BMI percentile (mean ± SD)	57.6	± 30.3	56.8	± 28.2	0.90
Comorbidity (at least 1)	29	(48%)	6	(20%)	0.01

	Mean	± SD	Mean	± SD	*p*
Initial curves					
Proximal thoracic (°)	15.6	± 7.3	10.9	± 6.9	0.009
Mid-thoracic (°)	30.1	± 10.9	24.1	± 6.9	0.02
Thoracolumbar / lumbar (°)	21.2	± 9.8	18.8	± 5.9	0.23
Maximum curve magnitude (°)	31.8	± 9.5	26.2	± 5.0	0.008
Maximum curve type (freq. (%))					0.41
Proximal thoracic	1	(2%)	1	(3%)	
Mid-thoracic	46	(75%)	21	(70%)	
Thoracolumbar / lumbar	8	(13%)	6	(20%)	
Maximal curve ≥ 30°	26	(47%)	4	(14%)	0.005
RVAD	8.5	± 7.8	8.8	± 8.8	0.88
Bracing characteristics					
Age at brace initiation (years; median(IQR))	7.8	± 1.44	8.2	± 1.3	0.21
Number braces (median (IQR))	3	(2–4)	2	2–3	0.45
Non-compliant	45	(74%)	8	(27%)	< 0.001
Brace-wear hours (median (IQR))	18	(18–20)	18	15–18	0.17
Bracing duration (years; mean ± SD)	4.7	1.93	4.5	2.0	0.67
Maximal curve magnitude in-brace (°)	17.2	± 8.2	12.4	± 5.4	0.009
Percent in-brace correction	55.3	± 23.8	64.4	± 23.6	0.11

### Non-operative follow-up

Of the 91 subjects, 30 (32%) had not undergone surgical intervention with greater than 2 years after brace completion (Fig. 1). Mean maximal curves were 22° ± 13.0° (5°–53°) at brace end, with 62% mid-thoracic, 28% proximal thoracic, and 11% thoracolumbar/lumbar curves for the non-operative group (Table 4). They were followed for a median of 3.3 years (2–10 years) with mean maximal curves at final follow-up of 23° ± 15.4° (2°–57°) (Table 4). Of those that presented with a curve between 26 and 30 degrees and did not require surgery, 93% had stabilization or improvement of their curve (Table 5). There were 18 patients that were released from care at the completion of bracing had a mean curve of 23° (6°–36°) and were not included in this analysis (Fig. 2).

**Table 4:** Curve characteristics over time for maximal curve at each time point and by curve type by surgical intervention group and for all subjects

Time point	Maximal curve			Proximal thoracic	Mid-thoracic	Thoracolumbar/lumbar	RVAD	
	*n*	Mean ± SD	Range	Mean ± SD	Mean ± SD	Mean ± SD	Mean ± SD	Range
Operative								
Presentation	57	32 ± 9.5	17–50	16 ± 7.3	30 ± 10.9	21 ± 9.8	9 ± 7.8	0–28
In-brace	60	17 ± 8.2	2–39	12 ± 7.5	15 ± 9.0	12 ± 7.7		
Brace end	61	53 ± 12.3	19–93	28 ± 10.8	51 ± 14.2	37 ± 14.3	14 ± 12.3	1–51
Preoperative	61	61 ± 12.1	38–93	28 ± 11.3	59 ± 13.1	39 ± 15.5		
Non-operative								
Presentation	28	26 ± 5.0	12–37	11 ± 6.9	24 ± 6.9	19 ± 5.9	10 ± 8.4	0–38
In-brace	29	12 ± 5.4	4–24	8 ± 5.9	8 ± 5.6	9 ± 5.7		
Brace end	30	22 ± 13.0	5–53	15 ± 11.3	20 ± 13.9	16 ± 7.8	7 ± 6.3	0–34
Last follow-up	25	23 ± 15.4	2–57	12 ± 11.4	22 ± 16.0	16 ± 10.4		
Entire cohort								
Presentation	83	30 ± 8.6	12–50	14 ± 7.4	28 ± 10.1	20 ± 8.7	9 ± 7.8	0–38
In-brace	87	16 ± 7.7	2–39	10 ± 7.2	13 ± 8.6	11 ± 7.2		
Brace end	91	43 ± 19.2	5–93	23 ± 12.5	41 ± 20.1	30 ± 16.0	14 ± 12.3	0–51

All values are reported in degrees (°)

*SD*, standard deviation; *RVAD* rib vertebral angle difference

**Table 5:** Distribution of curve magnitudes at presentation and final outcomes based on intervention group and SRS guidelines (*n* = 83)

Surgery		Curve outcome at the end of bracing / pre-surgery							
		Progression > 10°		Stabilization −10° to + 10°		Improvement > 10°		Final Curve ≥ 45°	
Initial curve	*N*	Freq.	(%)	Freq.	(%)	Freq.	(%)	Freq.	(%)
< 20°	3	3	(100%)	0	(0%)	0	(0%)	3	(3%)
20°–25°	13	12	(92%)	1	(8%)	0	(0%)	13	(12%)
26°–30°	13	12	(92%)	1	(8%)	0	(0%)	13	(12%)
31°–35°	8	8	(89%)	0	(0%)	0	(0%)	8	(8%)
36°–40°	5	4	(80%)	1	(20%)	0	(0%)	5	(4%)
41°–45°	6	4	(57%)	2	(29%)	0	(0%)	6	(4%)
46°–50°	7	6	(86%)	1	(14%)	0	(0%)	7	(6%)
Total *N*	55	49	(86%)	6	(11%)	0	(0%)	55	(49%)

Non-operative		Curve outcome after a minimum of 2 years follow-up							
		Progression > 10°		Stabilization − 10° to + 10°		Correction > 10°		Final Curve > 45°	
Initial curve	*N*	Freq.	(%)	Freq.	(%)	Freq.	(%)	Freq.	(%)
< 20°	1	0	(0%)	1	(100%)	0	(0%)	0	(0%)
20°–25°	10	1	(10%)	5	(50%)	4	(40%)	1	(10%)
26°–30°	13	1	(8%)	4	(31%)	8	(62%)	0	(0%)
31°–35°	2	1	(50%)	1	(50%)	0	(0%)	1	(50%)
36°–40°	2	0	(0%)	2	(100%)	0	(0%)	0	(0%)
41°–45°	0	0	(0%)	0	(0%)	0	(0%)	0	(0%)
46°–50°	0	0	(0%)	0	(0%)	0	(0%)	0	(0%)
Total *N*	28	3	(11%)	13	(46%)	12	(43%)	2	(7%)

### SRS brace outcome evaluation

The curve at presentation, grouped in 10° intervals with progression, were divided between those treated with surgery and brace only (Table 5). Of the 53 patients with curves < 30° at presentation, 29 patients (55%) underwent surgical intervention, and 24 did not. In the 30 that presented with a curve > 30°, 26 patients (87%) underwent surgery. Of the 83 patients with complete data, 52/83 (62%) progressed > 10°, 19/83 (23%) stabilized within 10°, and 12/83 (14%) improved > 10° at of the end of bracing or prior to surgical intervention (Table 5). Of those that did not undergo surgery, 3 (11%) progressed > 10°, 13/28 (46%) stabilized within 10°, and 12/28 (43%) improved > 10° with brace wear. Two curves (7% of the non-operative group) were > 45° at final evaluation.

## Discussion

This is a large group of children with JIS treated within a 10 year timeframe, consistent protocol, follow-up past skeletal maturity, and reporting adherent to the SRS and SOSORT guidelines [9, 21]. Although the SRS and SOSORT guidelines are specific to AIS, they are important in establishing common reported outcomes so that series can be compared and used in meta-analyses. This is imperative for less common conditions such as JIS.

We defined our JIS population as diagnosis of idiopathic scoliosis between 4 and 10 years old, but the lower threshold in the literature varies between 3 and 4 years [3, 4, 6, 8, 10–12, 19, 22]. Children under 4 years old may be treated with a Risser cast, and our group elected 4 years as the cutoff to create a standard treatment approach in JIS. Previous studies suggest that when JIS presents in children less than 6 years old, there is higher risk of progression and surgical intervention [5, 20]. Children braced under the age of 10 have reported surgical rate of 66%, versus children over age the age 10 is 25% [13]. Another report, however, found no difference in progression based on age under or over 8 years [14]. In our study, we did not identify age as a significant factor for curve progression and/or surgery.

The definition of “idiopathic” can also be debated. Some studies include children with epilepsy or mental deficiencies [8]. Yet others include operative intraspinal pathology (ISP), genetic conditions, ^3^2 cm leg length discrepancy and cardiac or mediastinal anomalies requiring thoracotomies [12]. We chose to include idiopathic epilepsy and non-operative ISP. Genetic conditions and cardiac abnormalities that required open heart surgery were excluded due to the association of these conditions with scoliosis. We found that non-operative ISP did not increase the risk for surgical intervention or progression compared to children without ISP.

Prior studies have shown that children with EOS have a high rate of intraspinal pathology (11–26%) [14–16, 18]. Our series also exhibits a high rate of ISP identified on MRI within the JIS population. In this series, 19/86 patients (22%) had non-operative MRI abnormalities which included 14 cases (16%) of ISP. Furthermore, there were 23 additional patients with ISP that required surgical intervention and were excluded from the study. Previous studies have demonstrated similar rates of operative ISP in their cohorts [12, 15–17]. We did not identify risk factors for ISP in our cohort.

The location of the primary scoliosis curve has been found to influence outcomes. Mid-thoracic curves are known to progress despite treatment, while deterioration is less frequently seen in non-thoracic curves [4, 6, 10, 12–14, 23, 24]. Studies with fewer thoracic curves and curves < 20° also report better outcomes of bracing [6, 19]. In this study, the location of the curve was not significant, although the most common location of the largest curve was mid-thoracic. Some authors speculate that initial curve magnitude is a risk factor for curve progression or poorer outcomes, with larger curves (^3^30°) trending toward progression [4, 11, 13, 19, 20, 24]. A higher initial curve Cobb angle and curves ^3^30° in our study had higher risk of progression, with 88% undergoing surgery. Proximal thoracic curves were also larger at presentation in our operative group (16° vs. 11°, *p* < 0.01). This combination of a proximal thoracic and large mid-thoracic curves may increase risk for curve progression to > 45° and need for surgery.

Few studies conform to the recommended SRS and SOSORT reporting guidelines when evaluating brace treatment for scoliosis [4, 6, 11, 13, 20]. These guidelines allow comparison and combination of studies into meta-analyses to increase study power and generalizability of treatment recommendations and outcomes. While they are described for AIS, the standard reporting is useful in rarer conditions such as JIS. In one study using these criteria, 28% of curves improved or had minimal change, with 8% curve resolution [4]. In the current literature, patient characteristics for curve progression > 5° include younger age at bracing, decreased in-brace correction, Lenke I and III curves, and curves < 20° [4, 11, 19]. Our study found that 52 subjects progressed more than 10° from the initial evaluation to the end of bracing or surgery, compared to 31 who stabilized or improved. Reported noncompliance was a significant factor in our study (OR 4.27, 95% CI 1.69–10.77, *p* = 0.002). One limitation of our case series is that compliance was based on parent and physician reporting. It is now our standard of care to use a temperature-sensitive compliance monitor in all braced patients.

The goal of bracing is to stop curve progression. Curve progression in similar cohorts range from 33 to 75%, with 50–60% undergoing surgical correction [5, 12]. This study was bias toward surgical correction as an end point, with lower follow-up rates for those that did not undergo surgical correction. Harshavardhana and Lonstein reported a 41% brace success rate using < 45° as the definition of success, however, at 2 years of post-brace follow-up, 64% were > 45° [13]. In our study, 62% experienced curve progression and 67% of curves underwent surgical correction. Of those that progressed, 94% underwent surgical correction. Studies by Aulisa et al. have an intention-to-treat analysis with 72% correction, 15% stabilization, 7% progression, and 7% surgery [6, 20]. These studies report the lowest surgical rate in the current literature and differ from the current study as they contain more lumbar and thoracolumbar curves, do not include curves presenting ^3^40°, and include an older patient population [6, 20]. Few studies suggest that curves ^3^40° can be stabilized with bracing [19, 25]. In our study, seven patients presented with curves from 36 to 40° with three not progressing and two not undergoing surgery. All patients presenting with curves > 40° underwent surgery at the average age of fusion 13 years. None of the patients underwent growing posterior constructs prior to the instrumented posterior fusion.

## Conclusion

This is a large series of JIS patients with a standard Boston bracing protocol followed to skeletal maturity. We found a high rate of intraspinal pathology, supporting routine attainment of MRI in children under the age of 10 years. The rate of JIS progression is high, with 63% progressing > 10° with Boston brace treatment. After at least 2 years of follow-up from skeletal maturity and Boston brace completion, the rate of curve correction or stabilization was 37%. In this retrospective case series, surgery was performed in 67% of patients braced for JIS, in 88% of patients with curve greater than 30°, and in 100% of patients with curve greater than 40°at initiation of bracing. Risk factors for curve progression are larger presenting curves and reported noncompliance to brace prescription of 18–20 h/day. All braced patients were able to not have posterior spinal instrumentation and fusion until skeletal maturity.
